# The Role of PTSD in Bi-directional Intimate Partner Violence in Military and Veteran Populations: A Research Review

**DOI:** 10.3389/fpsyg.2017.01394

**Published:** 2017-08-15

**Authors:** Gabriela Misca, Mary Ann Forgey

**Affiliations:** ^1^Institute of Health and Society, University of Worcester Worcester, United Kingdom; ^2^Graduate School of Social Services, Fordham University New York, NY, United States

**Keywords:** PTSD, IPV, military, veterans, domestic violence, couples, bi-directional IPV, combat-trauma

## Abstract

Evidence supporting the higher prevalence of PTSD linked to combat-related trauma in military personnel and veteran populations is well-established. Consequently, much research has explored the effects that combat related trauma and the subsequent PTSD may have on different aspects of relationship functioning and adjustment. In particular, PTSD in military and veterans has been linked with perpetrating intimate partner violence (IPV). New research and theoretical perspectives suggest that in order to respond effectively to IPV, a more accurate understanding of the direction of the violence experienced within each relationship is critical. In both civilian and military populations, research that has examined the direction of IPV's, bi-directional violence have been found to be highly prevalent. Evidence is also emerging as to how these bi-directional violence differ in relation to severity, motivation, physical and psychological consequences and risk factors. Of particular importance within military IPV research is the need to deepen understanding about the role of PTSD in bi-directional IPV not only as a risk factor for perpetration but also as a vulnerability risk factor for victimization, as findings from recent research suggest. This paper provides a timely, critical review of emergent literature to disentangle what is known about bi-directional IPV patterns in military and veteran populations and the roles that military or veterans' PTSD may play within these patterns. Although, this review aimed to identify global research on the topic, the majority of research meeting the inclusion criteria was from US, with only one study identified from outside, from Canada. Strengths and limitations in the extant research are identified. Directions for future research are proposed with a particular focus on the kinds of instruments and designs needed to better capture the complex interplay of PTSD and bi-directional IPV in military populations and further the development of effective interventions.

## Background

The wars in Iraq and Afghanistan have resulted in an unprecedented growth in research exploring the impact of war-zone deployment(s) on service members and their family functioning upon returning home. Among these issues, post-traumatic stress disorder (PTSD) has received extensive examination. Certain results have corroborated earlier findings involving Vietnam veterans and a consistent conclusion is that service members and veterans suffering from war-related PTSD have high prevalence rates of intimate partner violence (IPV) (e.g., Orcutt et al., 2003; Marshall et al., 2005; Taft et al., 2011; Smith et al., 2015; Trevillion et al., 2015). For example, in a recent systematic research review of military IPV prevalence, a 27.5% prevalence rate was found based on the studies reviewed of male veterans with PTSD who reported past year physical violence perpetration against female partners (Trevillion et al., 2015). This rate is substantially higher than the 12.7% IPV prevalence rate found in a nationally representative sample of participants with PTSD (Smith et al., 2015). While the majority of service members and veterans diagnosed with PTSD do not engage in IPV, military specific risk factors, such as length of deployment (McCarroll et al., 2003) and the type and level of combat exposure, including the killing of combatants and the witnessing of atrocities (e.g., Taft et al., 2005; Van Winkle and Safer, 2011) have been identified as factors that increase the risk of a service member or veteran with PTSD perpetrating IPV.

This body of research has increased understanding of which service members may be at risk of perpetration; however questions remain about the dynamic interplay of PTSD and IPV within the context of couple relationships. For example, the PTSD symptom of hyperarousal has been associated with IPV perpetration (Birkley et al., 2016); but how hyperarousal and other PTSD symptoms interact within the relationship context is not clear. Moreover, research highlighting correlations between combat-related PTSD and IPV has predominantly focused on male violence perpetration (Taft et al., 2005, 2009) and much less is known about the female service members' experience.

Equally important to gaining a fuller understanding of IPV and PTSD within the relationship context is the examination of the participation, if any, of the service member's spouse/partner in the violence. Civilian IPV research focused on understanding IPV directionality, as defined by the level and type of violence committed by each partner, identified bi-directional violence as the most prevalent pattern in both national and community samples (e.g., Capaldi and Owen, 2001; Caetano et al., 2005; Field and Caetano, 2005). Studies that further differentiated bi-directional violence found low-level bi-directional violence to be most prevalent (Capaldi and Owen, 2001; Williams and Frieze, 2005). Some studies have also identified unequal “asymmetrical” levels of violence or the primary aggressor within bi-directional patterns (Kernsmith, 2005; Temple et al., 2005; Williams and Frieze, 2005).

Military research that includes IPV directionality has been limited. In a methodological review, Rodrigues et al. (2015) identified seven studies that examined directionality with uni-lateral, bi-directional and asymmetrical patterns found (e.g., McCarroll et al., 2004; Chrysos et al., 2005; Forgey and Badger, 2006, 2010). Critically, less is known about the role of PTSD in these bi-directional patterns. Emerging findings suggest that the bi-directional pattern is the most prevalent when a service member has PTSD (Teten et al., 2009); moreover, the partner's use of aggression has been found to correlate with the service member IPV perpetration (LaMotte et al., 2015) and a service member's PTSD has also been identified as a risk factor for being a victim of IPV (Teten et al., 2009, 2010; LaMotte et al., 2014, 2015).

Complex typologies that go beyond describing the direction, type and level of IPV have been developed. Johnson and Ferraro (2000) proposed four patterns of violence organized by motivation; these include: intimate terrorism, violent resistance, mutual violence control and situational violence. While such typologies have greatly contributed to the understanding of various relationship contexts of IPV and have moved researchers and practitioners beyond thinking of IPV as a unitary phenomenon (Straus, 2011), their applicability to a military population is questionable due to the lack of consideration of specific military risk factors, including combat-related PTSD. More understanding about how these typologies fit or not within a military or veteran population is needed (Tinney and Gerlock, 2014). Of particular importance is a deeper understanding about the role of PTSD in IPV not only as a *risk factor for perpetration* but also as a *vulnerability factor for victimization*, as findings from recent research suggest (Teten et al., 2010; LaMotte et al., 2014, 2015).

While a significant body of research confirms the association between combat-related PTSD and IPV in military and veteran populations, examining in-depth the complex relationship of PTSD with the different patterns of IPV experienced by a military or veteran is critical. In light of the recent expansion of studies in this area, this timely review focuses specifically on what is known about IPV bi-directional patterns experienced in military and veteran populations and the roles that PTSD may play within these patterns.

## The review

This review systematically examined research on the role of service members' or veterans' PTSD in the IPV occurrence in couples comprising military service members or veterans involved in recent war conflicts. Searches of PsycINFO, MEDLINE, PILOTs, PubMed and Web of Science from 2003 to 2016 were performed. Searches included key words: (military/soldier^*^/arm^*^/combat/veteran^*^) AND (Iraq/Afghanistan/Enduring Freedom/Iraqi Freedom) and PTSD AND (IPV or [intimate partner AND (violence OR aggression)]. Further hand-searches were conducted on the bibliographies of the selected papers for other relevant articles and on papers that cited the selected studies.

Forty-four studies were retrieved and were individually assessed according to the following inclusion criteria: (1) reported data on both PTSD and IPV in couples of military personnel or veterans deployed to Iraq and Afghanistan; (2) included assessment of each partners' use of IPV (3) included assessment of the military personnel's PTSD (4) were peer-reviewed and reported in English. This systematic evaluation identified 8 studies (see Table 1). In light of the extensive heterogeneity of the studies (in terms of samples, design and measures used), and their findings (which precluded a thematical approach to analysis), a narrative synthesis was employed.

**Table 1:** ^a^Summary of studies included in review.

**Study (author, year)**	**Samples/war context**	**Respondents**	**Study design**	**PTSD & related measures**	**IPV & related measures**	**Main findings/evaluation**
**NON-US SAMPLES**						
Zamorski and Wiens-Kinkaid, 2013	1745 currently-serving Canadian Regular Forces members Post 9/11 (data collection: 2008)	Active duty member	Cross-sectional, population-based survey	4-item Primary Care PTSD Screen	Canadian General Social Survey (GSS) on Victimization (adapted from CTS)	Results indicate that 7% men engaged in bi-directional physical/sexual IP and 4.8% of women IPV not associated with recent deployments (not related to post-deployment reintegration) Identified patterns of bi-directional IPV but not analysed in relation to PTSD.
**US IRAQ AND AFGHANISTAN VETERANS**						
Finley et al., 2010	19 male veterans and 11 spouses	Couples	Cross sectional; 3 Case studies reports	16 veterans reported having been diagnosed with PTSD by at least one healthcare provider; 3 had no PTSD diagnosis.	Interviews	1 case of bilateral IPV Case studies suggest there may be distinct patterns of violence committed by PTSD-diagnosed veterans within the home; violence occurring in anger; “dissociative” violence.
LaMotte et al., 2015	65 male Iraq and Afghanistan veterans and their female partners	Couples	Cross-sectional	PTSD Checklist - PCL	CTS-2	Mutual IPV was the dominant pattern for veterans and partners Female partners were more likely to engage in one-sided physical IPV than the male veterans Female partner psychological IPV correlated with veterans PTSD; Veterans' PTSD symptoms may play a more prominent role in their partners' psychological IPV than the partners' own psychological distress; Evidence of PTSD as risk factor for veterans' IPV victimization. Unilateral patters analysed in relation to PTSD.
Teten-Tharp et al., 2016	100 male veterans and their female partners seeking relationship therapy	Couples	Cross-sectional	Primary or secondary diagnosis of PTSD in their medical record.	CTS-2	55 couples reported physical aggression, with 26 reporting mutual aggression, 19 one-sided female aggression and 8 one sided male aggression Male veterans reported perpetrating significantly more frequent sexual coercion than female partners reported perpetrating; Female partners reported perpetrating significantly more physical aggression than male veterans reported perpetrating; PTSD was not significantly associated with any form of violence perpetration or victimization and was equally distributed across patterns of violence.
**US VETERANS FROM MIXED WAR ERAS (INCLUDING IRAQ AND AFGHANISTAN VETERANS)**						
Gerlock et al., 2016	441 couples of male veterans and their partners Random sampling from a veteran population seeking treatment for PTSD Various wars incl. 14.7% Iraq and Afghanistan veterans	Couples	Cross-sectional mixed method study;	Clinician Administered PTSD Scale (CAPS)	ABI; Relationships Behavior Interview (RBI); + qualitative coding; CTS was used to asses childhood witnessing of inter-parental IPV; MPDQ	In 43% of veteran couples the male met the criteria for male perpetrated IPV; Of total sample 37% men reported being assaulted by their partners 34% women reported assaulting their partners—mostly in retaliation (bidirectional) Among veteran offenders, higher levels of abuse were correlated with lower levels of relationship mutuality. Evidence of predominant pattern of bi-directional IPV in veterans with PTSD but no further differentiation.
Wolf et al., 2013	286 couples from two VAs 90% veterans, male 36 spouses were also veterans 7 female-female same-sex couples. Various wars incl. 15.2% OEF/OIF Iraq and Afghanistan veterans	Couples	Cross-sectional	Clinician Administered PTSD Scale (CAPS) Structured diagnostic interviews as part of a larger study on PTSD and couple functioning; TLEQ	CTS-2	Both veteran and spouse trauma history and PTSD symptoms increased the risk of the veteran (but not the spouse) engaging in IP physical aggression Trauma history and PTSD symptoms account for both psychological and physical IP aggression No analysis of bi-directional IPV.
Teten et al., 2009	184 veteran couples seeking relationship therapy at a VA medical center Various war eras	Couples	Cross-sectional	All veterans had a primary diagnosis of PTSD	CTS and CTS-2	Patterns assessed: Nonviolent *n* = 81; one-sided female violent *n* = 24; one sided male *n* = 31; and mutually violent, *n* = 48 Male veterans with PTSD were overrepresented in the mutually violent group Higher incidence of violence among mutually violent female partners of veterans with PTSD than those whose male partners do not have PTSD The most violent individuals in the sample were found in mutual violent relationships. Evidence pf association PTSD and bi-directional IPV
Teten et al., 2010	94 veterans 59 Iraq and Afghanistan veterans 33 Vietnam veterans 2 both conflicts veterans	Male veterans	Cross-sectional	Routine diagnostic screening for PTSD at VA—not specified	CTS-2	Veterans with PTSD were significantly more likely to report psychologically abusing their partner than veterans without PTSD. All correlations between reported IPV reported and sustained were significant. Combat exposure did not distinguish individuals with and without PTSD Evidence to suggest bi-directional violence predominant but no analysis in relation to PTSD.

a *Abbreviations of measures used in the reviewed studies (see original source for citations): ABI, Abuse Behavior Inventory; Canadian General Social Survey (GSS) on Victimization (adapted from CTS); CAPS, Clinician Administered PTSD Scale; CTS, Conflict Tactics Scale; CTS-2, Conflict Tactics Scale Revised; Four-item Primary-Care PTSD Screen; MPDQ, Mutuality Psychological Development Questionnaire; PCL, PTSD Checklist; RBI, Relationships Behavior Interview; TLEQ, Traumatic Life Events Questionnaire*.

## Findings

The systematic searches identified one study that involved active duty service members in Canada and although the review aimed to identify global research on the topic, this was also the only study identified from outside the USA. Although, the focus of this review was on recent wars—i.e., conflict post 9/11—some of the studies included also veterans across older war eras. These studies were included in the review and discussed in terms of how their findings relate to recent war veterans.

The findings of studies included in the review are summarized in Table 1, and described in terms of the samples employed, including the war context, study design, and PTSD, IPV, and related measures; with the main findings evaluated and limitations highlighted. The studies were predominantly cross-sectional, with only two using mixed methods. As per inclusion criteria, all studies included a measure of military/veteran personnel's PTSD; however measure of PTSD for their partners'/spouses' were not employed across studies thus it is not possible to draw conclusions about the role of the partner's/spouse's PTSD in the couple's IPV.

Zamorski and Wiens-Kinkaid (2013) reported on a survey of IPV perpetration and victimization and their correlates, including PTSD symptoms, in a random sample of Canadian regular forces personnel (87.81% male). Results indicate that 7% of male military personnel and 4.8% of female military personnel reported engagement in bi-directional physical/sexual IPV. While the study employed a large, non-clinical sample of Canadian active duty male and female personnel, the choice of measures and analysis itself provided limited understanding about the dynamics between IPV and the military personnel's PTSD within the active duty personnel's intimate relationships. The Canadian General Social Survey (GSS) on Victimization was used to measure service members' self-reports of any acts of IPV committed by either member of the couple over the course of their entire relationship. The military personnel's current PTSD symptoms were measured using the four-item Primary Care PTSD screen; however the types of symptoms reported were not analyzed, nor was any information explored regarding the traumatizing event(s) that were the source of these symptoms. The analyses employed did not examine how particular PTSD symptoms or events relate to IPV patterns within the relationships; moreover it was difficult to ascertain the timeframe of the reported IPV which may have occurred years prior to current symptoms of PTSD.

Two mixed methods studies were identified, employing quantitative and qualitative assessments to capture the complexities of IPV and PTSD interactions in veteran samples. Finley et al. (2010) reported on three patterns of IPV that emerged from a study of families living with combat-related PTSD). Analyzing descriptions of IPV occurring between male veterans diagnosed with PTSD and their female partners, three cases studies are described that illustrate the distinct patterns found: violence committed in anger; dissociative violence; and parasomniac/hypnopompic violence which is violence due to hyperarousal during sleep. The case of violence committed in anger was a case of bi-directional violence, in which the partner responded with violence in retaliation. Despite the lack of generalizability, these findings provided a detailed description of the dynamic interplay of PTSD symptoms and IPV patterns in veterans.

In another mixed method study, Gerlock et al. (2016) compared (male) veterans (from a variety of wars (14.7% from Iraq and Afghanistan, 7.9% Persian Gulf, 59% Vietnam, 4.5% Korean and 0.9% World War II) in treatment for PTSD who have perpetrated IPV and those who have not. IPV perpetration was assessed by combining veteran and their (female) partner's reports via interviews and questionnaires, allowing an exploration of the type, level and direction of the violence including, if there was a primary aggressor and if the violence was motivated by retaliation. This study found a significant correlation between PTSD symptom severity and IPV and a high concordance in veteran and their partner's reports of IPV.

The strengths of this study emanate from the use of an in-depth mixed method assessment of PTSD severity and IPV, as well as an examination of perceived mutuality within a relationship and, importantly, secondary analyses of this data set found that mutuality mediated between PTSD symptom severity and IPV. The findings are limited, however, by the lack of exploring in more depth the motivational factors underlying the IPV beyond retaliation. And although PTSD symptom severity was examined, the role of PTSD symptoms themselves within each of the patterns was not, thus limiting the understanding of this dynamic. As certain types of combat exposure have been shown to increase the risk of IPV (Taft et al., 2005; Van Winkle and Safer, 2011), more examination of PTSD symptoms and the traumatic events underlying them is necessary to fully understand the dynamic interplay of PTSD and IPV in military and veteran populations, which becomes critical when samples combine veterans from across historical war eras, as in this study.

Among the quantitative studies identified, three involved veterans from different eras and two involved Iraq and Afghanistan veterans only. Wolf et al. (2013) examined the relationship between the veteran's PTSD and IPV in 296 couples of predominantly male veterans (majority from Vietnam and earlier wars, including 15.2% from Iraq and Afghanistan) and their female partners. The findings highlight that both veteran and spouse trauma history and PTSD symptoms increase the risk of the veteran but not the spouse engaging in IPV physical aggression; there was no relationship between any of the PTSD symptom-clusters and veteran perpetration of violence. Veteran combat exposure alone was not significantly correlated with physical or psychological aggression on the part of the veteran or spouse.

While each partner's use of violence in the last 6 months was explored, allowing an analysis of directionality, this analysis was not done and therefore the relationship of specific patterns of violence to PTSD was not examined. This is unfortunate since the study employed robust assessments of the veteran's PTSD, such as the Clinician Administered PTSD Scale (CAPS) to measure both the frequency and intensity of the PTSD symptoms within the past 6 months; and the Traumatic Life Events Questionnaire (TLEQ) to assess the type and level of traumatic events underlying the PTSD symptoms, allowing an exploration of the role, if any, of combat exposure in the violence perpetration. Although, no relationship was found between PTSD symptoms and combat exposure alone, this may be due to the majority of the participants being older Vietnam veterans whose combat exposure occurred decades ago and may have also experienced other types of trauma since.

Teten et al. (2009, 2010) examined the relationship between the veteran's PTSD and IPV in veterans and their partners seeking relationship therapy. In a sample of 184 couples involving veterans from various unspecified wars, Teten et al. (2009) identified three patterns of IPV: non-violent, mutually violent and one-sided violent. Veterans with a primary diagnosis of PTSD were overrepresented among couples reporting mutual violence. In a further sample of 94 Vietnam and Iraq and Afghanistan veterans, Teten et al. (2010) found that male Iraq and Afghanistan veterans with a PTSD diagnosis self-reported significantly more aggression toward their partner and also sustained more female perpetrated aggression than Iraq and Afghanistan veterans without PTSD or Vietnam veterans with PTSD.

While both of these studies report findings of high levels of mutual violence in veteran couples, there was limited analysis of the relationship between PTSD and these couples. Caution is needed as the data relied solely on veterans' self-reports of both IPV perpetration and victimization (Teten et al., 2009). Furthermore, all participants had a diagnosis of PTSD but there was no investigation of the symptom-clusters or the traumatic events that may be associated with the PTSD diagnosis. Consequently, little can be understood about the dynamic relationship between PTSD and IPV, other than the fact that a significant association was found.

Two studies reviewed focused exclusively on Iraq and Afghanistan male veterans and their female spouses. Teten-Tharp et al. (2016), in a sample of 100 couples, found 55 couples reporting physical aggression, with just over half reporting mutual aggression, and the rest reporting more one-sided female aggression than one-sided male aggression. Veteran's PTSD diagnosis, while prevalent within each pattern, was equally distributed and was not significantly associated with any specific pattern. Male veterans also reported perpetrating more frequent sexual coercion (operationalised as “*insisting on sex when the partner did not want it*”) than female partners reported; while female partners reported perpetrating more physical aggression than male veterans reported perpetrating. In the absence of any understanding of motivation or impact, which were not explored, the gender differences found must be interpreted with caution.

In another study, LaMotte et al. (2015) reported findings from 65 Iraq and Afghanistan veterans (recruited on the basis of their combat exposure but not required to have a PTSD diagnosis) and their partners regarding their IPV. The findings confirm mutual IPV as the dominant pattern but only the relationship of PTSD to the unilateral patterns of violence was analyzed. Female partners were found to perpetrate higher levels of physical IPA than the male veterans did, according to both veteran and combined reports; and female partner psychological IPV correlated with veterans' PTSD.

This study is important as it brings evidence that PTSD in Iraq and Afghanistan veterans may act as a *risk factor for IPV victimization* adding to our understanding of the complex relationship between combat-related PTSD and IPV. There are two potential explanations put forward for this interplay of PTSD's risk for perpetration and victimization: one refers to veteran's PTSD contributing to their IPV perpetration which in turn prompts partners' IPV as retaliation. The alternative explanation takes into account the carer's burden that veterans' PTSD places on their partners which in turn may lead to them react via IPV toward the veteran. The calculation of separate scores for the variety of physical acts of violence and for the frequency of the psychological violence acts allowed a more detailed understanding of who was doing what to whom in this study. However, the lack of enquiry into the motivation behind the violent acts committed (e.g., in control, defense, conflict) limited an understanding of these important dimensions. The severity of PTSD symptoms was measured and this allowed more understanding of their impact on the relationship; however, the specific symptom-clusters were not examined, nor was the specific traumatizing event, although all veterans in the study had been combat exposed.

## Conclusions and implications for future research

All but one of the studies reviewed confirmed through analyses bi-directional as the predominant pattern in veteran and, to some extent, in military active duty populations. However, collectively there was limited examination of the dynamic between PTSD and bi-directional IPV due to the lack of explicit analysis of this relationship and the limitations of measurements and samples that were employed.

Truly capturing the dynamics of PTSD in couples experiencing IPV requires the robust measurement of both PTSD and IPV for both military and their partner. For PTSD, this means exploring symptoms along with the underlying traumatic events (Wolf et al., 2013; Semiatin et al., 2017). While the findings of the studies reviewed provided varied understanding of the veteran's PTSD symptomatology and etiology, none of the studies explored PTSD on the part of the partner and the role that it may play in the bi-directional nature of the violence. This exploration is critical to understanding the dynamics of PTSD and IPV and the potential role that secondary stress may play in the partner's reciprocal violence (Renshaw et al., 2011).

For IPV, robust measurement must encompass all dimensions, including type, level, frequency, physical impact (e.g., injury), emotional impact (e.g., fear) and motives. While instruments exist to capture PTSD symptoms and underlying traumatic events (e.g., CAPS, TLEQ), there is not, to our knowledge, an instrument to reliably measure all dimensions of IPV. Until such an instrument is developed and validated, qualitative interviewing alongside the CTS2 might provide a suitable interim methodological solution.

When designing studies of the PTSD-IPV relationship, inclusion of other potential mediators identified in recent research, such as relationship mutuality (Gerlock et al., 2016), antisocial features (Taft et al., 2012) and social skills deficits (LaMotte et al., 2017) should be considered. The role of other military specific risk factors for IPV, for example, traumatic brain injury (TBI; Farrer et al., 2012) and substance abuse (Elbogen et al., 2014; Tinney and Gerlock, 2014) in the PTSD-IPV relationship also needs examination.

In addition to these measurement and design issues, attention must also be given to the populations from which the studies' samples are drawn. Most of the studies of veterans reviewed relied on samples recruited via Veteran Administration (VA), healthcare setting and clinical populations. Moreover, some studies included veterans from multiple war eras (e.g., Teten et al., 2009, 2010; Wolf et al., 2013; Gerlock et al., 2016) resulting in wide age range and large span of time in which the traumatic events possibly responsible for the PTSD symptoms may have occurred. Little has also been learned about the dynamics of combat-related PTSD in the intimate relationships of female active duty member and veterans and while many of the studies reviewed included female active duty members and veterans, they were a very small portion of the sample. Given the differences that may exist in terms of the female service members experience of both PTSD and IPV, separate studies are needed that focus on the female active duty and veteran population. When studying a military population attention must also be paid not only to the gender of the military member but also the military status of each spouse, since dual military couples may face unique challenges.

More robust research that reliably measures PTSD and IPV patterns for the purpose of analyzing this relationship and attends to the issues of sample selection bias is sorely needed to inform clinical decision making for military and veteran couples dealing with PTSD and IPV. In recognition that PTSD impacts the couple relationship (Dekel and Monson, 2010), conjoint treatment options for PTSD have been put forward (Monson et al., 2008, 2009), however, if and how these models are applicable when IPV is also involved has yet to be addressed (Williston et al., 2015). Future research that better explains the roles of PTSD as risk factor for IPV perpetration and victimization for each partner in military and veteran samples is essential to the development of safe and appropriate treatment options for these couples.

## Author contributions

Both authors contributed to literature searches and writing of this paper. Both authors approved the final manuscript.

### Conflict of interest statement

The authors declare that the research was conducted in the absence of any commercial or financial relationships that could be construed as a potential conflict of interest.
